# Neurophysiological Responses to Extra Virgin and Refined Olive Oils: A Pilot Study Combining Flavor Profiling, Electroencephalogram, and Standardized Low‐Resolution Brain Electromagnetic Tomography

**DOI:** 10.1002/fsn3.71636

**Published:** 2026-03-09

**Authors:** Hee Sung Moon, Se Young Yu, Hyeonjin Park, Younglan Ban, Ji Sun Kim, Eui‐Cheol Shin

**Affiliations:** ^1^ Department of GreenBio Science (BK21)/Food Science and Technology Gyeongsang National University Jinju Republic of Korea; ^2^ Agri‐Food Bio Convergence Institute Gyeongsang National University Jinju Republic of Korea

**Keywords:** commercial olive oil, EEG, standardized low‐resolution brain electromagnetic tomography, taste pattern, volatile compound

## Abstract

The flavor of olive oil is primarily influenced by minor compounds such as volatile and phenolic compounds, which play a significant role in consumer preferences. This study aimed to investigate the flavor profiles of two commercial olive oils: extra virgin olive oil (EVOO) and refined olive oil (ROO), using electronic sensors and gas chromatography–mass spectrometry (GC–MS) and to examine whether differences in flavor‐related profiles (volatile and phenolic/taste‐related indices) are accompanied by distinct EEG band responses and sLORETA source patterns during oral perception. Additionally, electroencephalogram (EEG) analysis and standardized low‐resolution brain electromagnetic tomography (*s*LORETA) were performed on healthy adults in their twenties to examine the neurophysiological responses. The results showed that EVOO exhibited richer green notes and more bitterness compared to ROO, which corresponded with distinct EEG activity patterns and *s*LORETA findings. Specifically, EVOO tended to activate a greater number of Brodmann areas than ROO, showing relatively higher activation in high‐beta and gamma waves. However, these findings should be interpreted as preliminary due to the limited sample size (*n* = 6) This pilot study suggests that EEG may capture subtle differences in brain activity associated with tasting EVOO and ROO, but larger blinded, placebo‐controlled studies are needed before firm conclusions can be drawn about neural correlates of oil flavor perception.

## Graphical abstract

This study presents an integrated chemosensory approach combining electronic nose and electronic tongue systems with chemometric analysis to characterize the sensory properties of refined olive oils. Multivariate modeling revealed clear discrimination among samples based on aroma and taste profiles. The results demonstrate the potential of sensor‐based analytics as an objective tool for food quality evaluation and sensory prediction.

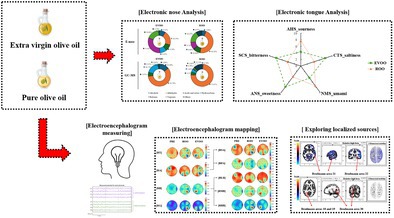

**Abbreviations**
EEG: electroencephalography
EVOO: extra virgin olive oil
RA: relative alpha wave
RB: relative beta wave
RFA: relative fast alpha wave
RG: relative gamma wave
RHB: relative high‐beta wave
RLB: relative low‐beta wave
RMB: relative mid‐beta wave
ROO: refined olive oil
RSA: relative slow alpha wave
RT: relative theta wave
*s*LORETA: standardized low‐resolution brain electromagnetic tomography

## 1 Introduction

Commercial olive oil is extracted directly from the fruit of the olive (*Olea europaea L*.) tree through mechanical crushing and pressing (Genovese et al. 2021). A staple in the Mediterranean diet, it is known for its nutritional benefits, mainly attributed to its phenolic compounds and unique sensory qualities (Infante et al. 2023). The aroma of olive oil is associated with various compounds, including aldehydes, alcohols, esters, hydrocarbons, ketones, and furans (Kalua et al. 2007). Specific compounds, including hexanal, *trans*‐2‐hexenal, *cis*‐3‐hexenal, and *trans*‐2‐pentenal, along with alcohols like *cis*‐3‐hexen‐1‐ol, *trans*‐2‐hexen‐1‐ol, and 1‐hexanol, contribute to the characteristic green notes of olive oil (Genovese et al. 2021; Kalua et al. 2007). These unique flavor attributes make olive oil highly appreciated by consumers worldwide (Harzalli et al. 2018).

Commercial olive oil grades are typically categorized based on a step‐by‐step process. Extra virgin olive oil (EVOO) is a natural product obtained through simple pressing without refining, while refined olive oil (ROO) undergoes a refining process (Gordillo et al. 2011; Fragaki et al. 2005). Refining is essential to neutralize free fatty acids present in commercial olive oil for ROO production (Ruiz‐Méndez et al. 2013). During this process, minor compounds (volatile and phenolic compounds) that contribute to the flavor properties of ROO may be lost or damaged (Genovese et al. 2021; Ruiz‐Méndez et al. 2013). These compounds play a key role in sensory perception, and their effects can be further investigated through neurophysiological responses to chemosensory stimulation (Hong et al. 2025).

These chemosensory properties of olive oils can be transmitted from the mouth to the nasal cavity via the retronasal route, enabling olfactory interaction (Genovese et al. 2021). Hong et al. (2025) found that chemosensory stimuli are processed in the human brain. Electroencephalogram (EEG) measurements and standardized low‐resolution electromagnetic tomography (*s*LORETA) analysis can offer insights into neural responses to chemosensory stimulation. Particularly, EEG captures changes in neurophysiological patterns across frequency bands, whereas *s*LORETA excels at identifying localized sources (Brodmann areas) and producing three‐dimensional (3D) images that standard EEG cannot generate (Hong et al. 2025; Lin et al. 2025). Therefore, the integrated application of these techniques is crucial for studying how flavor compounds influence neurophysiological responses.

In studies on flavor perception, holding food in the mouth without swallowing has been commonly used to specifically stimulate retronasal olfactory senses while reducing post‐ingestive and somatosensory effects (Xia et al. 2025; Yang et al. 2024). In this method, participants keep the sample in their oral cavity and breathe through the nose, which allows volatile compounds to reach the olfactory epithelium via the retronasal path. This setup allows for focused research on early chemosensory processing rather than full flavor perception, providing a solid foundation for linking retronasal stimulation to neural responses.

While there has been extensive research on neuroimaging of olfaction and gustation, most studies have focused on simple food matrices such as essential oils and the five basic tastes (García‐González et al. 2011; Pereira et al. 2025). Olive oil, a highly complex food with superior organoleptic properties, has been mainly studied for its functional aspects. Although some research has explored the flavor of olive oil, few studies have examined how refining processes affect its flavor profile. This study investigates the flavor differences in commercial olive oils based on refining processes, the neurophysiological responses to their perception in the oral cavity, and changes in localized brain activity (Brodmann areas). Biomimetic sensory‐based machine perception technology (electronic sensors) and gas chromatography–mass spectrometry (GC–MS) were applied for profiling the flavor properties, while EEG and *s*LORETA systems assessed neurophysiological responses and localized brain sources, respectively. This integrative approach represents a novel attempt to link olive oil flavor profiles with neural correlations, offering new insights into how refining influences both chemosensory perception and cognitive processing.

## 2 Materials and Methods

### 2.1 Experimental Samples

International EVOO (origin: Italy; acidity levels: 0.55%) and ROO (origin: Italy; acidity levels: 0.10%) products were purchased from a local grocery market (Jinju, Republic of Korea). The oils were classified based on the product labeling/specification provided by the manufacturer (EVOO vs. refined olive oil), supported by the reported free‐acidity values and the documented processing steps (with an explicit refining step for ROO). EVOO was produced from a blend of *Koroneiki*, *Manaki*, and *Tsounati* olive cultivars and processed in the following steps: raw material washing, crushing, malaxation, separation, filtration, storage, and packaging. ROO was produced using a blend of *Lecciana* and *Coratina* olive cultivars, undergoing raw material washing, crushing, malaxation, separation, refining, blending, filtration, storage, and packaging. The commercial olive oils were stored in the dark at room temperature (20°C–25°C) to minimize exposure to light.

### 2.2 Electronic Tongue (E‐Tongue) Analysis

The taste patterns of commercial olive oil products were analyzed using an E‐tongue (ASTREE II, Alpha MOS, Toulouse, France). The E‐tongue system consists of five electronic sensors and two indicator sensors related to the basic human perceptible tastes of sourness (AHS), saltiness (CTS), umami (NMS), sweetness (ANS), and bitterness (SCS). Olive oil (10 mL) was mixed with distilled water (90 mL) in an E‐tongue analysis vial, heated at 60°C on a stirring hot plate at 2264 g for 60 min to extract water‐soluble components. This oil–water extraction approach has been widely applied to hydrophobic matrices such as olive oil, ensuring efficient migration of hydrophilic compounds into the aqueous phase. After extraction, most of the upper oil layer was removed using a dropper, and the remaining samples were used for analysis. The samples were loaded into the sampler of the E‐tongue system, and the sensors were immersed in the sample solution for 2 min to measure the respective taste value intensities. To prevent contamination from contact between samples during the analysis process, each electronic sensor was rinsed in purified water after analysis. The analysis was performed with six replicates per sample (Ban et al. 2025; Moon et al. 2025).

### 2.3 Total Phenolic Content (TPC) Analysis

Each sample (1 mL) was added with ethanol (1 mL) and deionized water (5 mL), followed by the addition of 0.5 mL of Folin–Ciocalteu's reagent (Sigma‐Aldrich). After stirring for 5 min, 1 mL of 10% sodium carbonate (Na₂CO₃) solution was added. The mixture was centrifuged at 1764 g for 10 min. Then, 200 μL of the supernatant was transferred to a 96‐well microplate, and absorbance was measured at 750 nm using a UV–Vis spectrophotometer (Multiskan Go, Thermo Fisher Scientific). TPC was expressed in milligrams of gallic acid equivalents per milliliter of oil (mg GAE/mL) based on a calibration curve generated from gallic acid standards (0.01–0.08 mg/mL) (Yoon et al. 2024).

### 2.4 Electronic Nose (E‐Nose) Analysis

The volatile compounds of commercial olive oil products were analyzed using an E‐nose (HERACLES Neo, Alpha MOS). Here, 3 mL of each sample was placed in a headspace vial for analysis and stirred at 1200 g for 20 min at 40°C to saturate the vial with volatile compounds. The saturated volatile compounds were captured using an autosampler attached to the E‐nose, and 1000 μL of the captured volatile compounds were injected into the GC injection port equipped on the E‐nose using a syringe. The analytical conditions were set at a trap absorption temperature of 40°C, a trap desorption temperature of 250°C, an acquisition time of 110 s, and a hydrogen gas flow rate of 1 mL/min. The column used for the E‐nose analysis was an MXT‐5 column (Alpha MOS), and the oven temperature was maintained at 40°C for 5 s, then ramped up to 270°C at a rate of 4°C/s, and held for 30 s. To identify the volatile compounds of the aroma using the E‐nose system, the retention index based on carbon atoms was based on Kovat's index library, and the separated peak components were identified using the AcroChemBase (Alpha MOS) of E‐nose. All procedures were performed in three replicates per sample (Park et al. 2025).

For a visual summary of the volatile profile, the identified compounds were grouped into major volatile classes (e.g., aldehydes, ketones, alcohols, esters, acids, hydrocarbons). For the E‐nose data, the relative share (%) of each volatile group was calculated on a peak‐area basis as:

$$E-\text{nose group proportion}\;\left(\text{group}\;i,\%\right)=100\times \left(\Sigma \;\text{peak areas of compounds in group}\;i\right)/\left(\Sigma \;\text{peak areas of}\;\mathrm{all}\;\text{detected compounds}\right)$$

These normalized percentages indicate the proportion of each volatile group within the total E‐nose volatile profile of each sample.

### 2.5 GC–MS Analysis

The SPME–GC–MS analytical conditions were adapted, with minor modifications, from our previously published method (Moon et al. 2025). Briefly, the protocol was applied to the current instrument and samples, with slight adjustments to [e.g., SPME extraction time/temperature and/or GC oven temperature program] to achieve robust chromatographic separation and reproducible semi‐quantification under the present experimental setup. Solid phase microextraction (SPME; Supelco Inc., Bellefonte, PA, USA) fiber coated with 50/30 μm, divinylbenzene/carboxen/polydimethylsiloxane was used to collect the volatile compounds of the CFPs. The volatile compounds adsorbed on the SPME fiber were analyzed via GC–MS (Agilent 8890A and 5975C, Agilent Technologies, Santa Clara, CA, USA), and the column of the GC–MS was an SP −2560 column (L × I.D. 100 m × 0.25 mm, df 0.20 μm, Supelco Inc.). For the analysis, 3 mL of the sample was placed in a headspace vial and sealed with an aluminum cap, and the volatile compounds were equilibrated in the headspace for 5 min at 60°C. Subsequently, the SPME fiber was inserted into the vial and allowed to adsorb the volatile compounds for an additional 30 min. The adsorbed SPME fibers were then subjected to GC–MS analysis. This process was performed by an autosampler attached to the GC–MS. This analysis was conducted in duplicate, and it was possible to reduce the errors during the pre‐treatment process. The injector temperature was set to 220°C, and the oven temperature was initially maintained at 40°C for 5 min, followed by an increase to 200°C at a rate of 5°C/min. Helium was used as carrier gas at a 1.0 mL/min flow rate while operating in splitless mode. Each separated component in the total ionization chromatogram was identified using a NIST 12 mass spectral library integrated into the mass spectrum. The retention index (RI) was calculated for qualitative analysis using the n‐alkane retention time. Pentadecane (C15:0, Sigma‐Aldrich, St. Louis, MO, USA) is the internal standard, and the content (μg/kg) was calculated based on the peak areas of the analyzed volatile compounds in the sample relative to the peak area of the internal standard (pentadecane; 0.005 μg). For the GC–MS data, volatile compounds were semi‐quantified as contents (μg/kg) relative to the internal standard (pentadecane). To generate visual results, the relative share (%) of each volatile group was calculated using the semi‐quantified contents as:

$$\mathrm{GC}–\mathrm{MS}\;\text{group proportion}\;\left(\text{group}\;i,\%\right)=100\times \left(\Sigma \;\text{contents of compounds in group}\;i\right)/\left(\Sigma \;\text{contents of}\;\mathrm{all}\;\text{quantified volatile compounds}\right)$$

These normalized values provide a quantitative summary of the compositional distribution of volatile groups within each sample. Additionally, RI was determined according to Equation (1).

(1) $$\mathrm{RI}x=100n+100–\left(\left(\mathrm{tRx}–\mathrm{tRn}\right)/\left(\mathrm{tRn}+1–\mathrm{tRn}\right)\right)$$

where RI*x* is the RI of the unknown compound, *tRx* is the retention time of the unknown compound, *tRn* is the retention time of the *n*‐alkane, and *tRn* + 1 is the retention time of the next *n*‐alkane. *tRx* lies between *tRn* and *tRn* + 1 (*n* = number of carbon atoms) (Jo et al. 2025).

### 2.6 Characteristics of Participants and Experimental Designs

**FIGURE 1:**
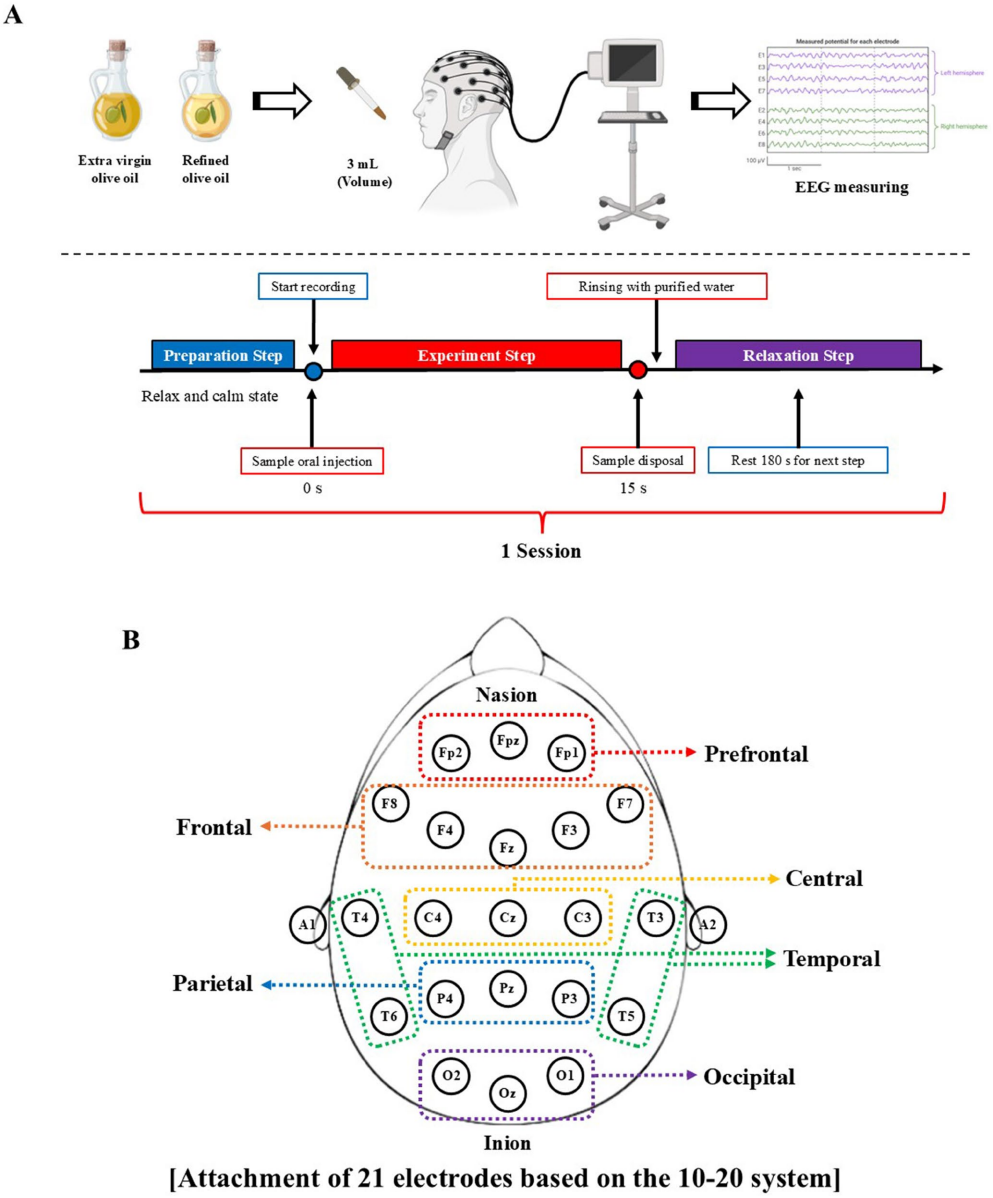
Experimental schematic diagram for EEG analysis (A), and placement of 21 electrodes based on the 10–20 system (B).

Six participants, including three males and three females, participated in the experiment. The inclusion criteria were non‐smoking participants, and the selected group consisted of six people in their twenties. Participants with low or impaired olfactory and gustatory abilities were excluded. Olfactory function was assessed using the Sniffin' Sticks test (Hummel et al. 2007), and gustatory function was assessed using Taste Strips (Landis et al. 2009). Only participants meeting normative criteria were included. The participants had no caffeine or alcohol intake in the 16 h preceding the experiment (Hong et al. 2025). Following a 10 min stabilization period for each participant, EEG measurements were conducted while holding olive oil in their oral cavity. A three‐milliliter dropper was used to administer olive oil into the participants' oral cavity. A schematic diagram of the EEG experiment is shown in Figure 1A. Participants were instructed to inhale through their nostrils, allowing the volatile compounds released from the oral cavity to reach the olfactory epithelium, thereby inducing the perception of orthonasal odor during EEG recording. All participants received detailed instructions, and they were provided with informed consent before starting the experiments. All human experimental procedures were approved by the Korea National Institute for Bioethics Policy IRB (approval No. P01–202101–13003) and were performed in accordance with the code of ethics for human experimentation of the World Medical Association (Declaration of Helsinki). Before participating in the study, informed consent was obtained from each subject.

### 2.7 EEG Recording

EEG measurements were recorded using an S‐24 EEG device (Biobrain Co., Daejeon, Republic of Korea). Twenty‐one‐disc electrodes were placed on the scalp using a paste, following the international 10–20 system (ElfixZ‐401 ce; Biobrain Co.) (Figure 1B). Electrodes were positioned across the prefrontal, frontal, temporal, central, parietal, and occipital regions. Additionally, reference electrodes (A1 and A2) were used in the EEG recording. The S‐24 EEG device (Biobrain Co.) has an amplifier input impedance greater than 1 GΩ, as specified by the manufacturer, which minimizes signal attenuation due to electrode–skin impedance. During electrode preparation, the electrode–skin impedance at all recording sites was maintained below 10 kΩ (typically 5–8 kΩ), in accordance with standard recommendations for reliable EEG acquisition. Participants closed their eyes during the EEG measurement, which began with a 15‐s baseline recording (control value), followed by a 15‐s recording during olive oil perception (experimental value). After each trial, the sample was expectorated, and the oral cavity was rinsed with purified water until no residual flavor remained. Three minutes later, the next sample was administered. The analog EEG signal data were converted to digital signals after being sampled at 250 Hz using an EEG program (Bioscan, Biobrain Co.). The converted signal data was then transmitted to a personal computer as a CSV file. The transmitted digital EEG data underwent filtering using the EEG program (Kim et al. 2018; Lee and Lee 2024). After filtering to remove artifacts through the EEG program, the EEG signals were categorized into delta (0–4 Hz), theta (4–8 Hz), alpha (8–13 Hz), beta (13–30 Hz), and gamma (> 30 Hz) waves, with experimental values expressed as the ratio to the control values (Hong et al. 2025). Brain topography was visualized using a brain mapping program (Bioscan Topomap; Biobrain Co.) based on mean EEG values from the participants. For the EEG mapping, paired *t*‐tests were conducted for each electrode and frequency band to evaluate relative power changes between treatments. Due to the small participant size and multiple comparisons, only a limited number of electrodes reached statistical significance; therefore, the topographies are represented as descriptive visualizations of spatial tendencies.

### 2.8 sLORETA Analysis

The *s*LORETA analysis was conducted based on EEG raw data, including the relative theta (RT), relative alpha (RA), relative beta (RB), relative gamma (RG), relative slow alpha (RSA), relative fast alpha (RFA), relative low‐beta (RLB), relative mid‐beta (RMB), and relative high‐beta waves (RHB). During the *s*LORETA procedure, a text file containing EEG data collected after perception of olive oils in the oral cavity was converted into a ‘slor’ file for each participant, followed by the execution of a corresponding sample test (paired group test, A‐A2 = B‐B2). In the *s*LORETA analysis, A2 and B2 were designated as eye‐close statuses, while a corresponding sample test was chosen to ascertain the difference in brain activation during oral perception of EVOO versus ROO in the mouth within the same participant. Statistical differences between voxels across all frequency bands were determined by projecting the statistical non‐parametric mapping (SnPM) method through 5000 random trials (simulation). The SnPM method was conducted in *s*LORETA using 5000 random permutations. The maximal statistic approach implemented in the software was applied to control the family‐wise error rate across all voxels. This procedure yields a permutation‐based critical threshold corresponding to a 5% family‐wise error rate (i.e., a conventional *α* = 0.05), rather than voxel‐wise exact *p*‐values. Only voxels whose test statistics exceeded this permutation‐derived threshold were considered to show significant differences between conditions. In this study, t‐values for the log‐transformed data were computed to ensure enhanced normality by minimizing the variance between the data. Among the t‐values calculated by repeating this process 5000 times, voxels with *t*‐values exceeding the threshold satisfying *p* < 0.05 were deemed to include the brain inside the localized sources, with statistically significant differences in EEG signals between the two groups (pre vs. EVOO, pre vs. ROO) (Hong et al. 2025; Kim 2023).

### 2.9 Hierarchical Cluster Analysis/ Heatmap

Multivariate analysis was conducted based on the EEG measurement's wavelength (raw data) value, and only wavelengths (RHB and RG) with significant differences in the results of *s*LORETA were chosen as raw data. Hierarchical cluster analysis (HCA) was applied to identify patterns and dendrograms between samples and variables using MetaboAnalyst 6.0 (https://www.metaboanalyst.ca) (Ban et al. 2025; Hong et al. 2025; Park et al. 2025). HCA was performed on the autoscaled data using Euclidean distance as the dissimilarity measure and Ward's minimum‐variance method (ward. D) as the linkage algorithm to generate the dendrograms and corresponding heatmaps.

## 3 Results and Discussion

### 3.1 Taste Pattern Results

**FIGURE 2:**
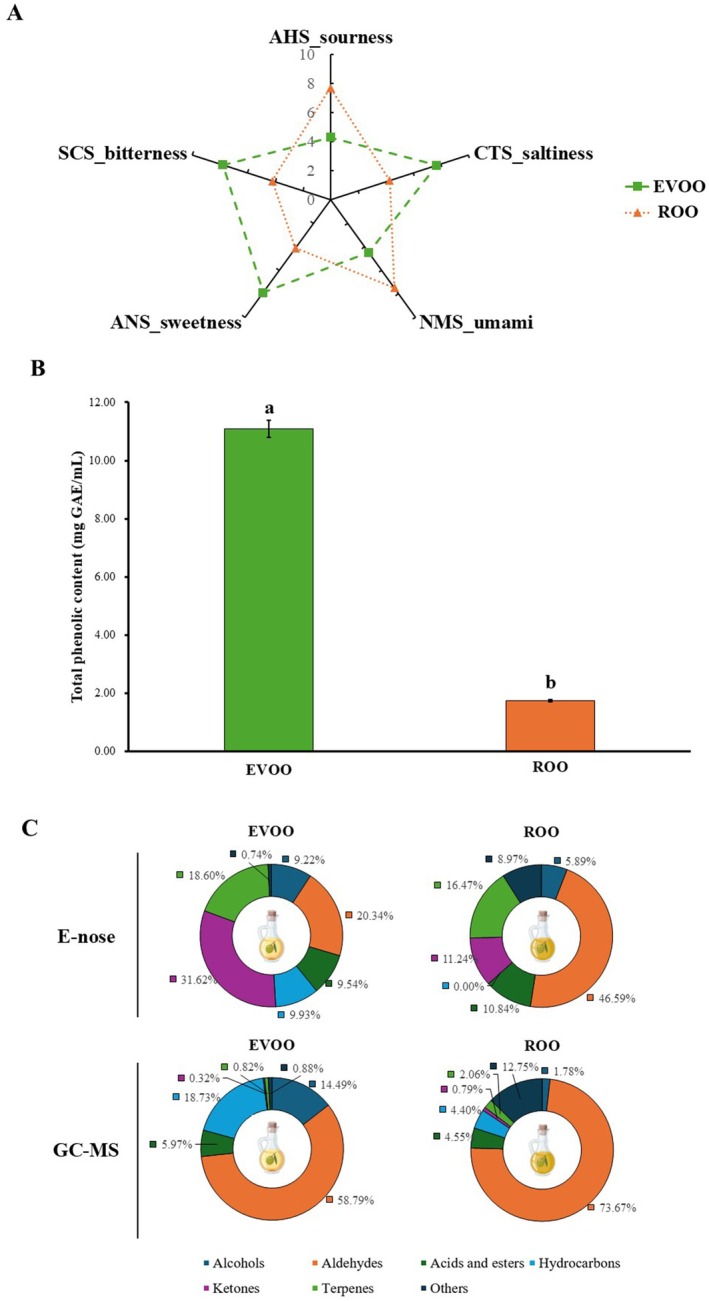
Taste profile of commercial olive oils (A), total phenolic content (TPC, indicated by different lowercase letters a–b to denote significant differences using Tukey's multiple range test, *p* < 0.05) (B), and volatile compounds composition analyzed by E‐nose and GC–MS percentages indicate the relative share of each volatile group within the total volatile profile (E‐nose, Peak‐area based; GC–MS, Semi‐quantified contents; μμg/kg, using pentadecane as an internal standard) (C).

Taste patterns of EVOO and ROO were analyzed using E‐tongue, and the results are presented in Figure 2A. The E‐tongue measures the sensor value for taste by detecting the reactivity of organic and inorganic substances in sensors related to the five tastes perceived by humans. The sensor reactivity was analyzed via taste screening (Lee et al. 2019). The analysis revealed that ROO had higher sensor values for sourness (AHS, 7.7) and umami (NMS, 7.5) than EVOO (AHS: 4.3; NMS: 4.5). In contrast, EVOO had higher sensor values for saltiness (CTS: 7.7 vs. 4.3), sweetness (ANS: 7.9 vs. 4.1), and bitterness (SCS: 7.8 vs. 4.2) compared to ROO (4.1). In addition, the sensor value for SCS was higher in EVOO (7.8) than in ROO. Thus, EVOO exhibited stronger intensities of sweetness, saltiness, and bitterness than ROO.

According to Esti et al. (2009), bitterness and pungency are key sensory characteristics of olive oil, which correlate with phenolic compounds. The comparison of TPC in EVOO and ROO (Figure 2B) showed that EVOO (11.08 ± 0.29 mg GAE/mL) had approximately 10 times higher TPC than ROO (1.75 ± 0.03 mg GAE/mL). This difference in TPC likely influenced the intensity of SCS in olive oil, leading to a significant difference in the E‐tongue analysis. Previous research suggests that the intensity of bitterness affects the perceived pleasantness of olive oil (Caporale et al. 2004). Therefore, the difference in bitterness intensity between EVOO and ROO is linked to TPC levels, which could affect consumer preferences. Trained expert panels typically describe high‐quality EVOO as defect‐free and often associated with green notes, bitterness, and pungency (Delgado and Guinard 2012). These oral sensations are important factors of consumer acceptance, and phenolic compounds—often reflected by total phenolic content (TPC)—contribute substantially to bitterness and pungency (Espejel et al. 2008; Krystallis and Ness 2005; Chousou et al. 2018). Therefore, the higher TPC observed in EVOO may be perceived as desirable “robustness” by some consumers but as excessive harshness by others; accordingly, TPC can influence preference in either a positive or negative direction depending on consumer segments and usage context.

### 3.2 Volatile Compounds Results

The percentage composition of volatile compounds in commercial olive oil is shown in Figure 2C as pie charts, and the detailed peak areas and contents are shown in Tables 1 and 2. The E‐nose identified three alcohols, two aldehydes, four hydrocarbons, four ketones, one terpene, and five other compounds in EVOO. Whereas two acids and esters, two alcohols, five aldehydes, one ketone, two terpenes, and one other compound were identified in ROO. Notably, ketones accounted for the highest proportion (31.62%; 9.81 peak area **×** 10^3^) of the total volatile compounds in EVOO, followed by aldehydes (20.43%; 6.31 peak area **×** 10^3^). In contrast, ROO exhibited the highest proportion of aldehydes (46.59%; 3.48 peak area **×** 10^3^), followed by terpenes (16.47%; 16.47 peak area **×** 10^3^). In addition, the GC–MS identified five alcohols, four aldehydes, five acids and esters, 14 hydrocarbons, one ketone, three terpenes, and three other compounds in EVOO. In addition, one alcohol, four aldehydes, three acids and esters, and four hydrocarbons, one ketone, two terpenes, and two other compounds in ROO. Notably, aldehydes accounted for the highest proportion (58.79%; 41264.83 μg/kg) of the total volatile compounds in EVOO, followed by hydrocarbons (18.73%; 13147.92 μg/kg). Also, ROO exhibited the highest proportion of aldehydes (73.67%; 12594.22 μg/kg), followed by other compounds (12.75%; 2178.88 μg/kg). In addition to sensory traits, the nutritional value of olive oil is primarily attributed to its high oleic acid content and minor components, while its aroma is significantly influenced by volatile compounds (Kalua et al. 2007). Importantly, the percentage values obtained by the E‐nose and GC–MS were calculated independently within each platform (i.e., relative peak‐area proportions of the compounds identified by that platform), and therefore are not directly comparable across methods. In particular, the ‘ketone’ proportion in the E‐nose reflects a grouped set of peaks assigned by the instrument library/column response, whereas under our GC–MS conditions, only one ketone was identified and quantified, resulting in a comparatively small compositional percentage.

**TABLE 1:** Volatile compounds of olive oils (EVOO and ROO) analyzed by E‐nose.

Compounds	RT (RI)	Sensory description^a^	Peak area × 10^3^	
			EVOO	ROO
Acids and esters (2)				
Methyl propanoate	24.06 (618)	Apple, Rum	ND	0.48 ± 0.61
Propyl heptanoate	75.09 (1200)	Apple, Sweet	ND	0.33 ± 0.20
Alcohols (4)				
Methanol	15.09 (436)	Pungent	1.21 ± 0.34	0.34 ± 0.09
2‐Penten‐1‐ol	39.82 (771)	Gasoline	0.22 ± 0.33	ND
3‐Hexen‐1‐ol	49.54 (860)	Green, Earthy	4.88 ± 5.87	ND
1‐Hexanol	49.65 (861)	Grassy, Green	ND	0.61 ± 0.74
Aldehydes (5)				
Propenal	16.33 (464)	Almond, Cherry	6.31 ± 0.82	3.13 ± 0.40
Butanal	20.67 (561)	Pungent, Musty	ND	0.21 ± 0.21
Hexanal	43.51 (802)	Grassy, Green	1.28 ± 1.41	0.71 ± 1.06
3‐Hexanal	43.77 (805)	Grassy, Green	ND	2.45 ± 3.62
Pentadecanal	99.49 (1718)	Fresh	ND	0.14 ± 0.09
Hydrocarbons (4)				
Butane	13.39 (398)	Faint	0.17 ± 0.06	ND
2‐Methyl hexane	27.06 (652)	—	0.08 ± 0.01	ND
3‐Methyl hexane	29.89 (683)	—	0.20 ± 0.08	ND
Nitrobenzene	71.02 (1132)	Almond, Oil	0.42 ± 0.23	ND
Ketones (5)				
Butan‐2‐one	22.43 (600)	Butter, Cheese	0.40 ± 0.15	ND
Ethyl maltol	76.69 (1229)	Candy	0.30 ± 0.07	ND
*γ*‐Decalactone	89.21 (1484)	Coconut, Sweet	ND	9.81 ± 8.79
*β*‐Ionone	89.41 (1489)	Floral, Powdery	5.68 ± 7.29	ND
2‐Hexadecanone	103.73 (1814)	Fruity	0.14 ± 0.06	ND
Terpenes (3)				
*γ*‐Terpinene	66.81 (1067)	Citrus, Fruity	0.09 ± 0.10	ND
Limonene oxide	71.31 (1137)	Citrus, Fresh	ND	1.15 ± 0.91
Thymol	80.13 (1293)	Earthy, Spicy	ND	0.08 ± 0.02
Others (6)				
Diethyl ether	17.55 (491)	Etheral	2.69 ± 0.57	ND
Ethanethiol	18.71 (517)	Earthy	0.24 ± 0.30	ND
2,2‐Dichloropropane	23.65 (614)	—	0.16 ± 0.14	ND
1‐Butanamine	25.04 (629)	Ammoniacal	0.07 ± 0.07	ND
2‐Acetylpyridine	64.61 (1036)	Biscuit, Bread	ND	0.67 ± 0.49
1,8‐Cineole	65.21 (1044)	Mentholic	0.14 ± 0.15	ND

Abbreviations: EVOO, extra virgin olive oil; ND, not detected; RI, retention index; ROO, refined olive oil; RT, retention time.

^a^ Sensory description: These were obtained from AcroChemBase (Alpha MOS) for E‐nose library annotations and supported by published literature (Cecchi et al. 2021; Yan et al. 2020) on olive oil volatile compounds and associated odor notes.

**TABLE 2:** Volatile compounds of olive oils (EVOO and ROO) analyzed by GC–MS.

Volatile compounds	RT (min)	RI	Mean ± SD (μg/kg)		I. D.
			EVOO	ROO	
Alcohols (5)					
3‐Methyl‐4‐Penten‐1‐ol	9.702	819	610.74 ± 863.72	ND	MS
3‐Hexen‐1‐ol	14.282	958	9122.49 ± 121.01	304.98 ± 431.31	MS
Cycloheptanol	15.31	987	359.03 ± 29.37	ND	MS
2‐Nonen‐1‐ol	17.488	1058	21.28 ± 30.10	ND	MS
2,13‐Octadecadien‐1‐ol	20.805	1169	54.60 ± 77.22	ND	MS
Aldehydes (5)					
Hexanal	7.612	< 800	3891.82 ± 58.31	2603.74 ± 62.44	MS/RI
2‐Hexenal	9.255	804	31489.09 ± 684.83	9216.11 ± 272.69	MS/RI
2,4‐Hexadienal	11.201	865	949.93 ± 438.73	ND	MS
2‐Heptenal	12.682	907	ND	270.28 ± 382.23	MS
Nonanal	17.254	1050	4933.99 ± 86.22	504.09 ± 29.10	MS
Acids and esters (7)					
Pentanoic acid	3.405	< 800	ND	16.32 ± 23.07	MS
Hexanoic acid	13.487	933	832.47 ± 79.55	745.14 ± 86.92	MS
Acetic acid	14.466	963	2950.12 ± 22.93	ND	MS
Benzoic acid	17.019	1043	267.59 ± 16.19	ND	MS
Carbonic acid	22.727	1238	50.94 ± 72.04	ND	MS
Oxalic acid	25.304	1335	ND	17.14 ± 24.24	MS
3,4‐Dihydroxymandelic acid	31.595	1596	87.68 ± 124.00	ND	MS
Hydrocarbons (17)					
1,3‐Pentadiene	6.749	< 800	675.31 ± 37.35	ND	MS
1,4‐Pentadiene	6.932	< 800	128.50 ± 181.72	ND	MS
Ethylidenecyclopropane	7.03	< 800	175.90 ± 248.76	ND	MS
2,4‐Hexadiene	9.742	820	ND	550.36 ± 778.33	MS
1‐Methyl‐cyclohexene	11.238	866	314.93 ± 445.38	ND	MS
3‐Ethyl‐1,5‐octadiene	12.018	888	1065.22 ± 690.95	67.79 ± 95.87	MS
3,4‐Dimethyl‐2‐hexene	12.758	909	4604.55 ± 268.32	ND	MS
3‐Ethyl‐1,5‐octadiene	13.935	947	1749.00 ± 2473.46	ND	MS
Cyclopropane	15.911	1005	224.46 ± 317.44	ND	MS
3,8‐Dimethyl‐decane	20.01	1142	ND	57.79 ± 81.73	MS
1‐Tridecene	20.187	1148	3723.56 ± 5265.91	ND	MS
3‐Octadecene	20.363	1154	46.59 ± 65.89	ND	MS
Cyclopropane	21.746	1200	72.26 ± 10.11	ND	MS
Tetradecane	25.298	1400	21.79 ± 30.81	ND	MS/RI
*α*‐Farnesene	27.987	1442	100.44 ± 142.04	ND	MS/RI
Heptacosane	30.022	1528	2.86 ± 4.05	ND	MS
2,5,6‐Trimethyl‐octane	34.93	1752	ND	15.61 ± 22.08	MS
Ketones (2)					
6‐Methyl−5‐hepten‐2‐one	13.651	938	222.23 ± 314.28	ND	MS
1,7‐Octadien‐3‐one	17.624	1062	ND	134.92 ± 190.80	MS
Terpenes (4)					
Isoprene	6.885	< 800	337.53 ± 477.34	ND	MS
Limonene	14.955	977	ND	285.53 ± 25.24	MS/RI
*β*‐Ocimene	15.556	994	199.40 ± 281.99	66.97 ± 94.71	MS
Cedrene	27.622	1427	37.99 ± 53.72	ND	MS
Other compounds (5)					
3‐Methyl‐phenol	15.131	982	257.12 ± 363.62	ND	MS
1‐Methylene‐1H‐indene	19.63	1128	ND	23.95 ± 33.86	MS
5‐O‐Methyl naringenin	23.492	1260	ND	2154.93 ± 3047.53	MS
Copaene	24.853	1317	336.52 ± 21.79	ND	MS/RI
11‐Decyl‐docosane	32.191	1624	25.18 ± 35.61	ND	MS

Abbreviations: EVOO, extra virgin olive oil; ND, not detected; RI, retention index; ROO, refined olive oil; RT, retention time.

According to previous studies, the characteristic green and fruity aroma of olive oil is primarily attributed to volatile compounds such as aldehydes and alcohols found in fresh olive oil. For example, aldehydes such as hexanal and 3‐hexanal, as well as alcohols like 3‐hexen‐1‐ol and 1‐hexanol, play important roles in this aroma (Genovese et al. 2021). In E‐nose and GC–MS analysis, 3‐hexen‐1‐ol, which is associated with grassy notes, was detected exclusively in EVOO, with a notably high peak area (4.88 ± 5.87) and content (9122.49 ± 121.01) (Tables 1 and 2). In Table 1, among the aldehydes, the peak area of hexanal in EVOO was nearly twice as high as in ROO, whereas 3‐hexanal (2.45 ± 3.62) was detected only in ROO. Since hexanal and 3‐hexanal are formed through different enzymatic oxidation pathways (Sabatini and Marsilio 2008), these differences may reflect variations in the refining process.

In the E‐nose results, the total percentage composition of aldehydes was twice as high in ROO (46.59%) as in EVOO (20.34%) (Figure 2C). Additionally, aldehydes associated with green and grassy notes, such as hexanal and 3‐hexanal, showed higher individual peak areas in ROO. On the other hand, the percentage composition of alcohol was significantly higher in EVOO (9.22%) compared to ROO (5.89%), and 3‐hexen‐1‐ol (4.88 ± 5.87) showed the most prominent difference. Analysis of volatile compounds using GC–MS also identified that 3‐hexen‐1‐ol and hexanal, detected by the E‐nose, were commonly found in EVOO at relatively higher concentrations than in ROO (Table 2). 3‐Hexen‐1‐ol was detected at a concentration of 9122.49 ± 121.01 in EVOO, but only 304.98 ± 431.31 in ROO. Hexanal was detected at a concentration of 3891.82 ± 58.31 in EVOO, but at a concentration of 2603.74 ± 62.44 in ROO. The percentage composition derived from the E‐nose and GC–MS results should be interpreted within each platform, as the detected peak sets and response characteristics differ; thus, cross‐platform comparisons of class percentages (e.g., ketones) are semi‐quantitative.

These results suggest that key volatile compounds contributing to green notes, such as hexanal and related C6 compounds, are likely lost during the refining process (García et al. 2006; Ruiz‐Méndez et al. 2013). Therefore, the refining process not only alters the chemical composition of olive oil but also significantly affects its flavor profile, particularly the green and bitter sensory attributes characteristic of high‐quality EVOO. Refining is intended to improve stability and remove undesirable odors by reducing odor‐active volatiles and minor polar components, especially during deodorization. Consequently, refined olive oils typically show a less complex headspace profile and attenuated “green/fruity” notes relative to extra virgin olive oils, which retain a richer volatile composition from mechanical extraction (Yan et al. 2020). In parallel, refining markedly reduces phenolic constituents that contribute to bitterness and pungency, thereby shifting the sensory profile toward a milder taste (Lucci et al. 2020). These processing‐driven sensory changes provide a mechanistic context for the lower phenolic‐related indices and simplified aroma‐related patterns observed for ROO in the present study.

### 3.3 Effects of Olive Oil Perception on EEG Activity

**FIGURE 3:**
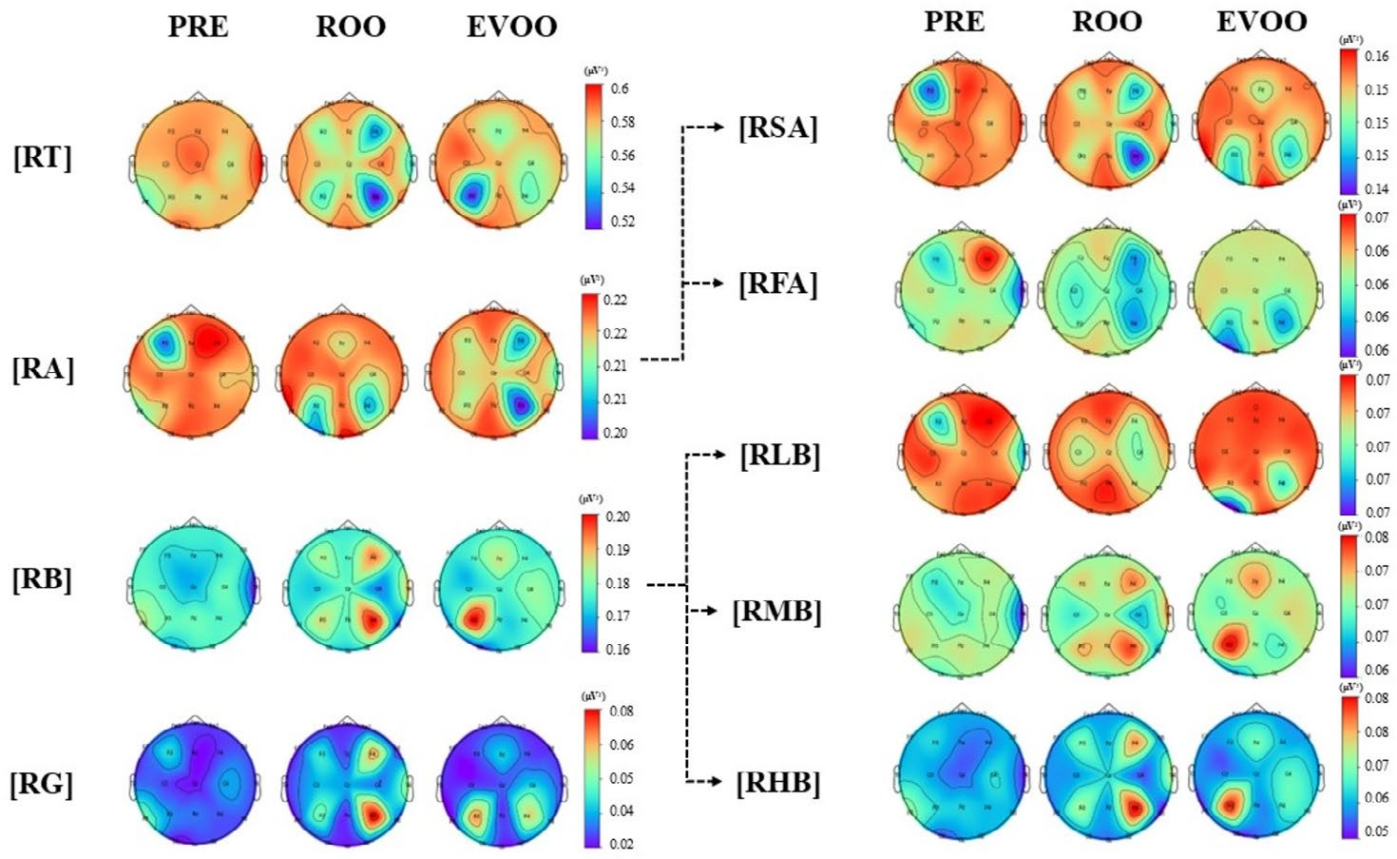
EEG signal changes during oral perception of commercial olive oils across different frequency bands: RT (relative theta), TA (relative alpha), RB (relative beta), RG (relative gamma), RFA (relative fast alpha), RSA (relative slow alpha), RLB (relative low‐beta), RMB (relative mid‐beta), and RHB (relative high‐beta) waves.

In this study, changes in the four major EEG frequency bands (theta [4–8 Hz], alpha [8–13 Hz], beta [13–30 Hz], and gamma [> 30 Hz] waves) were measured. The results of EEG mapping (topography) are shown in Figure 3. EEG activity and topography results are based on 21 EEG electrodes classified into six main regions: prefrontal, frontal, temporal, central, parietal, and occipital.

In the EEG mapping results of oral perception with ROO, the relative theta wave (RT) exhibited a decreasing tendency in the right frontal (F4) and parietal lobes (P4) during ROO perception. Similarly, the relative alpha (RA) wave showed a decreasing tendency in the left frontal (F3), parietal (P3), occipital (O1), and right parietal lobes (P4) during ROO perception. In contrast, the relative beta (RB) and relative gamma (RG) waves demonstrated an increasing tendency in the right frontal (F4) and parietal lobes (P4) during ROO perception. In this study, a further subdivision of the significant EEG frequency bands revealed that both relative fast‐ (RFA; 11–13 Hz) and low‐ (RSA; 8–11 Hz) alpha waves exhibited a decreasing tendency in the right frontal (F4) and parietal lobes (P4) during ROO perception, consistent with the findings for the RT. On the contrary, the mid‐ (RMB; 15–20 Hz) and high‐beta (RHB; 20–30 Hz) waves exhibited an increasing tendency in the right frontal (F4) and parietal lobes (P4). Whereas low‐beta (RLB; 13–15 Hz) waves showed no noticeable tendency.

In the EEG mapping results of oral perception with EVOO, the RT exhibited a decreasing tendency in the left parietal lobes (P3). On the contrary, in the RA, the right frontal (F4) and parietal lobes (P4) showed a decreasing tendency during EVOO perception. In RB, the left parietal lobe (P3) showed an increased tendency during EVOO perception. The RG is also similar to the RB tendency. In more detailed subdivisions of RA into RSA and RFA, the left and right parietal lobes (P3, P4) exhibited a decreasing trend following the perception of EVOO. On the contrary, the RMB and RHB exhibited an increasing tendency in the left parietal lobes (P3). Whereas RLB showed a noticeable decreasing tendency in the left occipital lobe (O1).

The wavelength‐specific EEG reflects the sum of electrical activities in the brain; each of these specific EEGs is associated with a particular mental status (Hong et al. 2025). For example, RMB is activated during attention and concentration, RHB is activated during tension and stress, and RG is activated during a high degree of cognitive action (learning, concentration, and memorization) (Hong et al. 2025; Kang et al. 2013). In this study, both EVOO and ROO elicited an increase in RMB and RHB, indicating heightened attention, tension, and cognitive activity. However, the cortex regions activated by each oil differed. ROO predominantly activated the right frontal (F4) and parietal (P4) lobes, whereas EVOO primarily activated the left parietal lobe (P3). Accordingly, the EEG topographies in Figure 3 should be interpreted as illustrating spatial tendencies in relative power rather than statistically robust differences at each scalp site, whereas the main inferential conclusions were drawn from the *s*LORETA analysis.

Volatile compounds of olive oils are substances of low molecular weight, and some can reach the olfactory epithelium, where they dissolve in mucus and bind to olfactory receptors to create odor sensations (Kalua et al. 2007). In addition, taste perception occurs on the tongue, where the taste buds are located in three types of papillae. When taste substances stimulate receptors, they trigger a cascade of reactions via G protein‐coupled pathways (gustatory perception). Similarly, stimulation of receptors by volatile compounds leads to transmission of nerve impulses by releasing these compounds in the oral cavity (olfactory perception) (Tuorila and Recchia 2014; Hong et al. 2025). The observed differences in brain waves between EVOO and ROO correspond to their distinct phenolic and volatile compound profiles. However, since no explicit correlation or regression analysis was performed, these results should be interpreted as suggesting potential associations rather than direct causation by specific compounds. To establish statistical associations between chemical composition and neurophysiological responses, future studies are needed involving multiple oils with stepwise adjustments in the concentration levels of phenolic and key volatile compounds, along with larger participant samples.

### 3.4 Multivariate Patterning of EEG Features and Source Localization (HCA/Heatmap and sLORETA)

**FIGURE 4:**
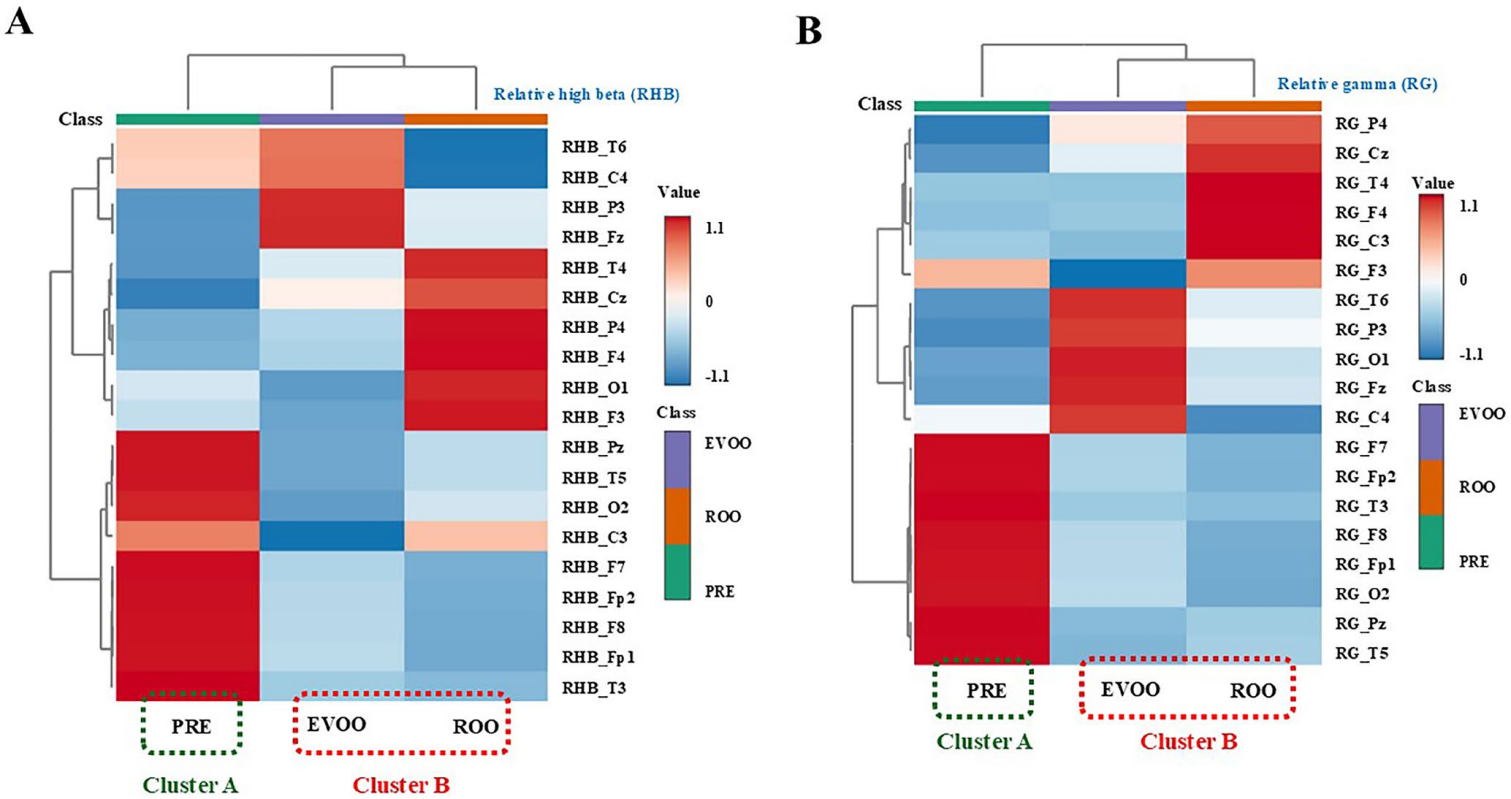
Hierarchical clustering analysis (HCA) and heatmap based on relative high‐beta (RHB) (A) and relative gamma (RG) (B) EEG features. In both bands, clustering primarily separated the pre‐stimulation condition (PRE) from the stimulation conditions (EVOO and ROO), indicating systematic multivariate changes in higher‐frequency activity during oral perception of olive oil.

The HCA/heatmap results based on relative high‐beta (RHB) and relative gamma (RG) features are shown in Figure 4. In both bands, hierarchical clustering primarily separated the pre‐stimulation (PRE) condition from the stimulation conditions (EVOO and ROO), indicating systematic multivariate modulation of higher‐frequency EEG features during oral perception of olive oil.

**FIGURE 5:**
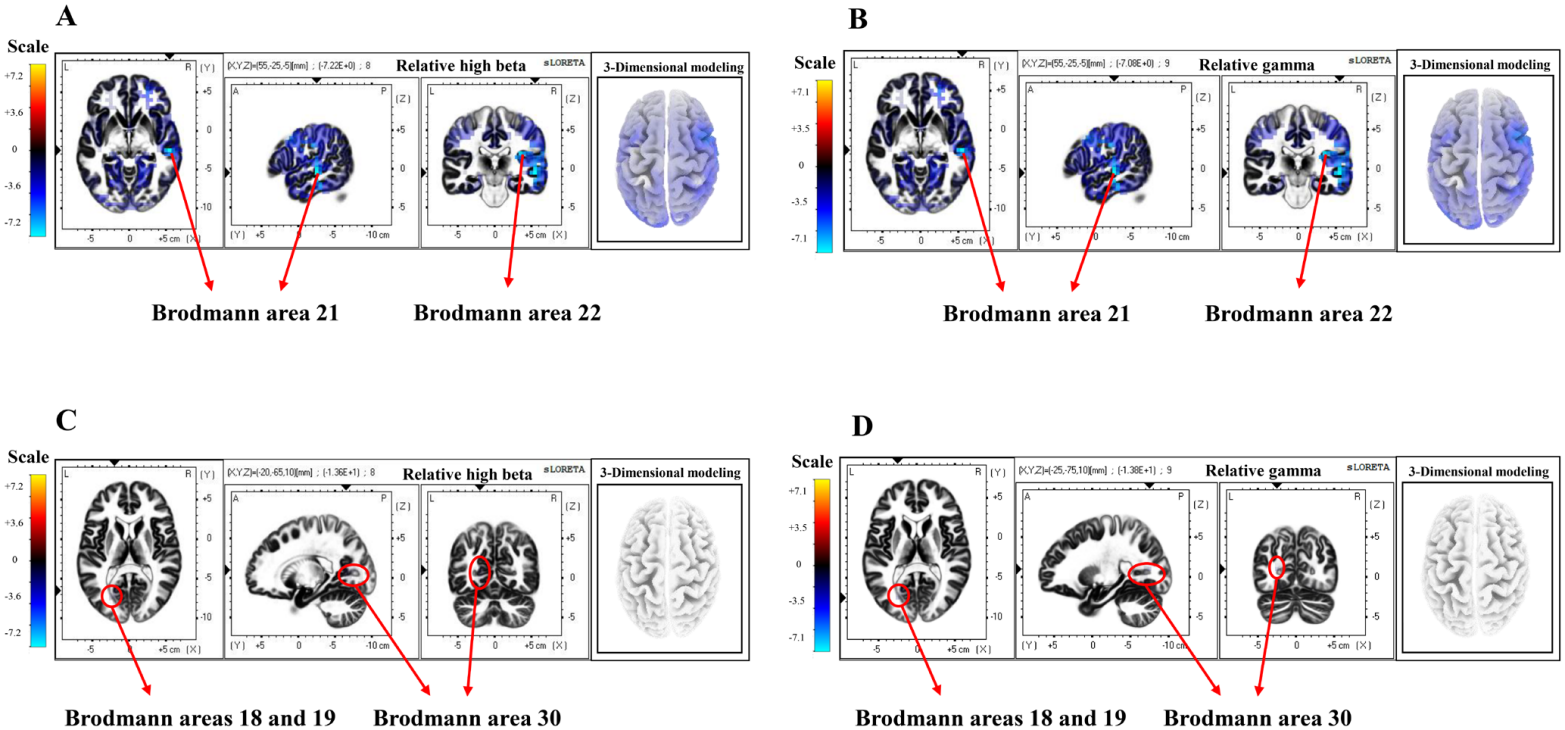
Localized source results for RHB (relative high beta) and RG (relative gamma) waves in the ROO (A, B) and EVOO groups (C, D) using sLORETA.

Because scalp EEG has limited spatial specificity due to the ill‐posed inverse problem (Brinkmann 2024), we applied standardized low‐resolution brain electromagnetic tomography (sLORETA) to estimate distributed cortical current density from the EEG data. sLORETA analyses were performed for PRE, ROO, and EVOO, and the resulting source maps are shown in Figure 5A–D and Table 3. Source‐level contrasts were computed by subtracting PRE voxel values from ROO and EVOO voxel values, respectively, and analyses were restricted to the RHB and RG bands, where significant effects were observed. To summarize the most prominent source differences, we report maxLOR values as in Kim (2023). maxLOR was used to indicate the voxel showing the largest (most pronounced) source difference in each contrast.

**TABLE 3:** Changes in localized brain signal sources by olive oil perception in the oral cavity.

Type	Brain wave	MNI coordinates			Voxel value	Brodmann area	Lobe	Structure
		*X*	*Y*	*Z*				
ROO	Relative high beta	55	−25	−5	−7.22	21	Temporal lobe	Superior temporal gyrus
		55	−25	0	−6.91	22		
	Relative gamma	55	−25	−5	−7.08	21	Temporal lobe	Superior temporal gyrus
EVOO	Relative high beta	−20	−70	10	−11.7	18	Occipital lobe	Cuneus gyrus
		−25	−80	−15	−10.2			Lingual gyrus
		−25	−80	−20	−9.74	19	Occipital lobe	Fusiform gyrus
		−25	−75	−10	−9.21			Lingual gyrus
		−20	−65	10	−13.6	30	Limbic lobe	Posterior cingulate gyrus
		−20	−65	5	−12.2			
		−20	−70	5	−9.22			
		−25	−75	10	−12.3		Occipital lobe	Cuneus gyrus
	Relative gamma	−20	−70	10	−10.3	18	Occipital lobe	Cuneus gyrus
		−25	−80	−15	−9.2			Lingual gyrus
		−25	−80	−20	−9.21	19	Occipital lobe	Fusiform gyrus
		−25	−75	10	−13.8	30	Occipital lobe	Cuneus gyrus
		−20	−65	10	−13.3		Limbic lobe	Posterior cingulate gyrus
		−20	−65	5	−11.5			
		−25	−70	10	−9.36			

Abbreviations: EVOO, extra virgin olive oil; ROO, refined olive oil.

In this study, the superior temporal gyrus was found to be activated in the RHB and RG during oral perception of ROO. The superior temporal gyrus (Brodmann areas 21 and 22) is functionally linked to connecting lower‐level auditory processing structures with higher‐level association areas involved in abstract aspects of language (Yi et al. 2019). In contrast, during oral perception of EVOO, *s*LORETA revealed increased current density in the posterior cingulate, cuneus, lingual gyrus, and fusiform gyri, mainly corresponding to Brodmann areas 18, 19, and 30 in the high‐beta and gamma bands compared to PRE. The cuneus and lingual gyri are known to support non‐visual functions such as language processing and memory when visual sensation is blocked (Palejwala et al. 2021). Additionally, the posterior cingulate gyrus is considered one of the least understood regions of the cerebral cortex (Foster et al. 2023). However, Foster et al. (2023) suggested that the posterior cingulate gyrus may help integrate internal (non‐sensory or post‐sensory) information and influence ongoing learning and decision‐making. These regions, part of the visual association and retrosplenial cortices, have been linked to visual associative processing, contextual memory, and multisensory integration (Strotzer 2009). Nonetheless, given the exploratory nature of the current study and its small sample size, these source‐localization results should not be seen as definitive evidence for specific higher‐order cognitive functions but rather as tentative indications that chemosensory processing of EVOO might engage distributed visual–associative, contextual networks.

Overall, these source‐localization findings suggest that EVOO and ROO are associated with distinct cortical activation patterns in higher‐frequency bands during oral perception.

## 4 Conclusion

This study explored the flavor profiles of two types of olive oils (EVOO and ROO) produced by different refining processes using biomimetic sensory‐based machine perception techniques (electronic sensors) and GC–MS. To investigate neurophysiological responses during oral perception of olive oil, EEG activity was recorded and cortical sources were examined using source‐localization analysis (sLORETA).

EVOO and ROO exhibited distinct sensory characteristics, particularly in taste‐ and odor‐related profiles, and were associated with different cortical activation patterns in the high‐beta and gamma bands under our experimental conditions. These sensory differences are consistent with processing‐related changes in volatile compounds contributing to “green‐note” aroma (e.g., hexanal and related C6 compounds) and with the role of phenolic constituents in bitterness and pungency.

At the source level, EVOO (relative to PRE) showed broader activation involving posterior cingulate and occipital/association regions (BA 18/19/30), whereas ROO involved superior temporal regions (BA 21/22). Although high‐frequency EEG activity has been linked to integrative and attention‐related processing in other contexts, the present pilot data should be interpreted cautiously and does not justify definitive attribution to specific higher‐order cognitive functions.

This exploratory study provides preliminary evidence that tasting EVOO and ROO is associated with different patterns of high‐beta and gamma EEG activity in a small group of participants; however, the present data do not establish generalizable “neural correlates of oil flavors” in a broad sense.

Limitations of this study include the lack of blinded tasting and placebo control, the small sample size (*n* = 6), and the restricted sampling of olive oils. Specifically, we tested only two commercial products (one EVOO and one ROO). Therefore, the observed differences may reflect product‐ or batch‐specific characteristics (e.g., cultivar, origin, processing conditions, storage history) rather than category‐level effects. Moreover, because the tested oils did not cover a broad range of sensory notes (e.g., varying intensities of green/fruity aroma and bitterness/pungency), the generalizability of the observed flavor–EEG relationships across diverse olive oils is limited. In addition, this pilot study did not include trained sensory panel data; therefore, direct quantification of perceived sensory attributes and sensory–instrument correlations could not be established. Library‐derived sensory descriptors (AcroChemBase, Alpha MOS) were used for instrumental annotation and should be interpreted as proxies rather than human sensory outcomes.

Future research using larger and more diverse cohorts, rigorously randomized and blinded placebo‐controlled designs, and multiple EVOO and ROO samples spanning diverse cultivars/brands and sensory profiles will be necessary to confirm and extend these observations. Future studies should also incorporate trained sensory profiling and multiblock data‐fusion approaches (e.g., PLS regression and/or MFA) to directly link sensory attributes and chemical markers to EEG‐derived measures.

## Author Contributions

**Hee Sung Moon:** conceptualization (equal), methodology (equal), writing – original draft (equal). **Se Young Yu:** formal analysis (equal), methodology (equal). **Hyeonjin Park:** formal analysis (equal), methodology (equal). **Younglan Ban:** formal analysis (equal), methodology (equal). **Ji Sun Kim:** formal analysis (equal), methodology (equal). **Eui‐Cheol Shin:** conceptualization (equal), funding acquisition (equal), methodology (equal), supervision (equal), writing – review and editing (equal).

## Funding

This work was supported by National Research Foundation of Korea, NRF‐2022R1I1A3066192.

## Conflicts of Interest

The authors declare no conflicts of interest.

## Data Availability

Data will be made available on request.

## References

[fsn371636-bib-0001] Ban, Y. , H. Park , S. J. Hong , S. Y. Yu , H. S. Moon , and E. C. Shin . 2025. “Sensomics and Chemometrics Approaches of Differential Brewed and Roasted Coffee (*Coffea Arabica*) From Ethiopia Using Biomimetic Sensory‐Based Machine Perception Techniques: Effects of Caffeine on Bitter Taste and the Generation of Volatiles.” Food Chemistry 476: 143407. 10.1016/j.foodchem.2025.143407. 39977987

[fsn371636-bib-0002] Brinkmann, B. H. 2024. “Technical Considerations in EEG Source Imaging.” Journal of Clinical Neurophysiology 41, no. 1: 2–7. 10.1097/WNP.0000000000001029. 38181382 PMC12702581

[fsn371636-bib-0003] Caporale, G. , S. Policastro , and E. Monteleone . 2004. “Bitterness Enhancement Induced by Cut Grass Odorant (Cis‐3‐Hexen‐1‐Ol) in a Model Olive Oil.” Food Quality and Preference 15, no. 3: 219–227. 10.1016/S0950-3293(03)00061-2.

[fsn371636-bib-0004] Cecchi, L. , M. Migliorini , and N. Mulinacci . 2021. “Virgin Olive Oil Volatile Compounds: Composition, Sensory Characteristics, Analytical Approaches, Quality Control, and Authentication.” Journal of Agricultural and Food Chemistry 69, no. 7: 2013–2040. 10.1021/acs.jafc.0c07744. 33591203

[fsn371636-bib-0005] Chousou, C. , E. Tsakiridou , and K. Mattas . 2018. “Valuing Consumer Perceptions of Olive Oil Authenticity.” Journal of International Food & Agribusiness Marketing 30, no. 1: 1–16. 10.1080/08974438.2017.1382418.

[fsn371636-bib-0006] Delgado, C. , and J. X. Guinard . 2012. “Internal and External Quality Mapping as a New Approach to the Evaluation of Sensory Quality–A Case Study With Olive Oil.” Journal of Sensory Studies 27, no. 5: 332–343. 10.1111/joss.12000.

[fsn371636-bib-0007] Espejel, J. , C. Fandos , and C. Flavián . 2008. “The Influence of Consumer Degree of Knowledge on Consumer Behavior: The Case of Spanish Olive Oil.” Journal of Food Products Marketing 15, no. 1: 15–37. 10.1080/10454440802470565.

[fsn371636-bib-0008] Esti, M. , M. Contini , E. Moneta , and F. Sinesio . 2009. “Phenolics Compounds and Temporal Perception of Bitterness and Pungency in Extra‐Virgin Olive Oils: Changes Occurring Throughout Storage.” Food Chemistry 113, no. 4: 1095–1100. 10.1016/j.foodchem.2008.08.076.

[fsn371636-bib-0010] Foster, B. L. , S. R. Koslov , L. Aponik‐Gremillion , M. E. Monko , B. Y. Hayden , and S. R. Heilbronner . 2023. “A Tripartite View of the Posterior Cingulate Cortex.” Nature Reviews Neuroscience 24, no. 3: 173–189. 10.1038/s41583-022-00661-x. 36456807 PMC10041987

[fsn371636-bib-0011] Fragaki, G. , A. Spyros , G. Siragakis , E. Salivaras , and P. Dais . 2005. “Detection of Extra Virgin Olive Oil Adulteration With Lampante Olive Oil and Refined Olive Oil Using Nuclear Magnetic Resonance Spectroscopy and Multivariate Statistical Analysis.” Journal of Agricultural and Food Chemistry 53, no. 8: 2810–2816. 15826023 10.1021/jf040279t

[fsn371636-bib-0012] García, A. , M. V. Ruiz‐Méndez , C. Romero , and M. Brenes . 2006. “Effect of Refining on the Phenolic Composition of Crude Olive Oils.” Journal of the American Oil Chemists' Society 83, no. 2: 159–164. 10.1007/s11746-006-1189-8.

[fsn371636-bib-0013] García‐González, D. L. , J. Vivancos , and R. Aparicio . 2011. “Mapping Brain Activity Induced by Olfaction of Virgin Olive Oil Aroma.” Journal of Agricultural and Food Chemistry 59, no. 18: 10200–10210. 10.1021/jf202106b. 21838262

[fsn371636-bib-0014] Genovese, A. , N. Caporaso , and R. Sacchi . 2021. “Flavor Chemistry of Virgin Olive Oil: An Overview.” Applied Sciences 11, no. 4: 1639. 10.3390/app11041639.

[fsn371636-bib-0015] Gordillo, B. , L. Ciaccheri , A. G. Mignani , M. L. Gonzalez‐Miret , and F. J. Heredia . 2011. “Influence of Turbidity Grade on Color and Appearance of Virgin Olive Oil.” Journal of the American Oil Chemists' Society 88, no. 9: 1317–1327. 10.1007/s11746-011-1787-y.

[fsn371636-bib-0016] Harzalli, U. , N. Rodrigues , A. C. Veloso , et al. 2018. “A Taste Sensor Device for Unmasking Admixing of Rancid or Winey‐Vinegary Olive Oil to Extra Virgin Olive Oil.” Computers and Electronics in Agriculture 144: 222–231. 10.1016/j.compag.2017.12.016.

[fsn371636-bib-0017] Hong, S. J. , S. Yoon , Y. Ban , et al. 2025. “Investigation of Non‐Volatile and Volatile Compound Profiles in Arabica Coffee Extracts and Neurophysiological Effects of Different Chemosensory Stimulation According to Sex: Insights From Electronic Sensors, EEG, and sLORETA Approaches.” Food Chemistry 467: 142211. 10.1016/j.foodchem.2024.142211. 39642421

[fsn371636-bib-0018] Hummel, T. , G. Kobal , H. Gudziol , and A. J. E. A. Mackay‐Sim . 2007. “Normative Data for the “Sniffin'sticks” Including Tests of Odor Identification, Odor Discrimination, and Olfactory Thresholds: An Upgrade Based on a Group of More Than 3,000 Subjects.” European Archives of Oto‐Rhino‐Laryngology 264, no. 3: 237–243. 10.1007/s00405-006-0173-0. 17021776

[fsn371636-bib-0019] Infante, R. , M. Infante , D. Pastore , et al. 2023. “An Appraisal of the Oleocanthal‐Rich Extra Virgin Olive Oil (EVOO) and Its Potential Anticancer and Neuroprotective Properties.” International Journal of Molecular Sciences 24, no. 24: 17323. 10.3390/ijms242417323. 38139152 PMC10744258

[fsn371636-bib-0020] Jo, S. M. , H. S. Moon , S. J. Hong , et al. 2025. “Exploring Flavor Patterns in the Peel of Tangor: A New Citrus Variety Based on Electronic Sensors and GC–MS/O.” Food Chemistry 485: 144415. 10.1016/j.foodchem.2025.144415. 40288347

[fsn371636-bib-0021] Kalua, C. M. , M. S. Allen , D. R. Bedgood Jr. , A. G. Bishop , P. D. Prenzler , and K. Robards . 2007. “Olive Oil Volatile Compounds, Flavour Development and Quality: A Critical Review.” Food Chemistry 100, no. 1: 273–286. 10.1016/j.foodchem.2005.09.059.

[fsn371636-bib-0022] Kang, S. Y. , M. K. Kim , and H. W. Ryu . 2013. “Influence of the Concentration of Lavender Oil on Brain Activity.” Korean Journal of Aesthetic and Cosmetology 11, no. 6: 1099–1107. http://e‐ajbc.org/journal/view.php?number=680.

[fsn371636-bib-0023] Kim, D. S. , Y. M. Goo , J. Cho , et al. 2018. “Effect of Volatile Organic Chemicals in *Chrysanthemum indicum* Linné on Blood Pressure and Electroencephalogram.” Molecules 23, no. 8: 2063. 10.3390/molecules23082063. 30126122 PMC6222417

[fsn371636-bib-0024] Kim, J. S. 2023. “Comparative Analysis of Brain Activity During Performing Number Sense and Mental Rotational Tasks: An EEG Study Using sLORETA.” Brain, Digital, & Learning 13, no. 4: 587–611. 10.31216/BDL.20230034.

[fsn371636-bib-0025] Krystallis, A. , and M. Ness . 2005. “Consumer Preferences for Quality Foods From a South European Perspective: A Conjoint Analysis Implementation on Greek Olive Oil.” International Food and Agribusiness Management Review 8, no. 2: 62–91. 10.22004/ag.econ.8161.

[fsn371636-bib-0026] Landis, B. N. , A. Welge‐Luessen , A. Brämerson , et al. 2009. ““Taste Strips”—A Rapid, Lateralized, Gustatory Bedside Identification Test Based on Impregnated Filter Papers.” Journal of Neurology 256, no. 2: 242–248. 10.1007/s00415-009-0088-y. 19221845

[fsn371636-bib-0027] Lee, J. , D. S. Kim , J. Cho , et al. 2019. “ *Perilla Frutescens* Britton: A Comprehensive Study on Flavor/Taste and Chemical Properties During the Roasting Process.” Molecules 24, no. 7: 1374. 10.3390/molecules24071374. 30965657 PMC6479574

[fsn371636-bib-0028] Lee, O. , and H. Lee . 2024. “Development and Printing of Three‐Dimensional Electrodes for the High Body Adhesion of Smart Wear.” Fashion and Textiles 11, no. 1: 29. 10.1186/s40691-024-00392-w.

[fsn371636-bib-0029] Lin, S. , Y. Du , Y. Xia , et al. 2025. “Changes of Food‐Cue Processing in Major Depressive Disorder Patients With Decreased Appetite: An Event‐Related Potential Study.” Appetite 210: 107939. 10.1016/j.appet.2025.107939. 40179445

[fsn371636-bib-0030] Lucci, P. , V. Bertoz , D. Pacetti , S. Moret , and L. Conte . 2020. “Effect of the Refining Process on Total Hydroxytyrosol, Tyrosol, and Tocopherol Contents of Olive Oil.” Food 9, no. 3: 292. 10.3390/foods9030292. PMC7143469 32150867

[fsn371636-bib-0031] Moon, H. S. , S. Y. Yu , Y. Ban , et al. 2025. “Sensomics Combined With Chemometrics Approaches of Enzymatically Hydrolyzed Animal By‐Product Proteins Using Biomimetic Sensory‐Based Machine Perception Techniques and Gas Chromatography‐Olfactometry‐Mass Spectrometry (GC‐O‐MS).” Food Chemistry: X 26: 102343. 10.1016/j.fochx.2025.102343. 40123869 PMC11930180

[fsn371636-bib-0032] Palejwala, A. H. , N. B. Dadario , I. M. Young , et al. 2021. “Anatomy and White Matter Connections of the Lingual Gyrus and Cuneus.” World Neurosurgery 151: e426–e437. 10.1016/j.wneu.2021.04.050. 33894399

[fsn371636-bib-0033] Park, H. , Y. Ban , S. J. Hong , et al. 2025. “Antioxidant and Chemosensory Properties of Rice ( *Oryza sativa* L.) Bran Under Different Oven‐Roasting Conditions.” Food Chemistry 476: 143496. 10.1016/j.foodchem.2025.143496. 39987805

[fsn371636-bib-0034] Pereira, D. R. , H. R. Pereira , M. L. Silva , P. Pereira , and H. A. Ferreira . 2025. “Impact of Five Basic Tastes Perception on Neurophysiological Response: Results From Brain Activity.” Food Quality and Preference 131: 105572. 10.1016/j.foodqual.2025.105572.

[fsn371636-bib-0036] Ruiz‐Méndez, M. V. , M. R. Aguirre‐González , and S. Marmesat . 2013. “Olive Oil Refining Process.” In Handbook of Olive Oil, edited by R. Aparicio and J. Harwood . Springer US. 10.1007/978-1-4614-7777-8_19.

[fsn371636-bib-0037] Sabatini, N. , and V. Marsilio . 2008. “Volatile Compounds in Table Olives ( *Olea Europaea* L., Nocellara del Belice Cultivar).” Food Chemistry 107, no. 4: 1522–1528. 10.1016/j.foodchem.2007.10.008.

[fsn371636-bib-0038] Strotzer, M. 2009. “One Century of Brain Mapping Using Brodmann Areas.” Clinical Neuroradiology 19, no. 3: 179–186. 10.1007/s00062-009-9002-3. 19727583

[fsn371636-bib-0039] Tuorila, H. , and A. Recchia . 2014. “Sensory Perception and Other Factors Affecting Consumer Choice of Olive Oil.” Olive Oil Sensory Science: 55–80. 10.1002/9781118332511.ch3.

[fsn371636-bib-0040] Xia, X. , Y. Cheng , Z. Zhang , et al. 2025. “Advancing Research on Odor‐Induced Sweetness Enhancement: A EEG Local‐Global Fusion Transformer Network for Sweetness Quantification Combined With EEG Technology.” Food Chemistry 463: 141533. 10.1016/j.foodchem.2024.141533. 39388878

[fsn371636-bib-0041] Yan, J. , M. Alewijn , and S. M. van Ruth . 2020. “From Extra Virgin Olive Oil to Refined Products: Intensity and Balance Shifts of the Volatile Compounds Versus Odor.” Molecules 25, no. 11: 2469. 10.3390/molecules25112469. 32466443 PMC7321329

[fsn371636-bib-0042] Yang, T. , P. Zhang , J. Hu , et al. 2024. “Exploring the Neural Correlates of Fat Taste Perception and Discrimination: Insights From Electroencephalogram Analysis.” Food Chemistry 450: 139353. 10.1016/j.foodchem.2024.139353. 38636376

[fsn371636-bib-0043] Yi, H. G. , M. K. Leonard , and E. F. Chang . 2019. “The Encoding of Speech Sounds in the Superior Temporal Gyrus.” Neuron 102, no. 6: 1096–1110. 10.1016/j.neuron.2019.04.023. 31220442 PMC6602075

[fsn371636-bib-0044] Yoon, S. , H. Jeong , S. J. Hong , et al. 2024. “Oven‐Roasting Effects the Fatty Acid Composition, Antioxidant Properties, and Oxidative Stability of Pomegranate (*Punica Granatum* L.) Seed Oil.” Preventive Nutrition and Food Science 29, no. 2: 190. 10.3746/pnf.2024.29.2.190. 38974588 PMC11223916

